# Barriers and strategies in detection and management of elevated Lipoprotein(a) in hospital: A pre-implementation qualitative study of cardiology healthcare professionals

**DOI:** 10.1371/journal.pone.0333789

**Published:** 2025-10-15

**Authors:** Wann-Jia Loh, Linh Thai, Bik-Ling Poon, Jonathan Yeo, Jian-Jing Tan, Elaine Lum

**Affiliations:** 1 Department of Endocrinology, Changi General Hospital, Singapore, Singapore; 2 Duke-NUS Medical School, Singapore, Singapore; 3 Health Services & Systems Research, Duke-NUS Medical School, National University of Singapore, Singapore, Singapore; 4 Department of Pharmacy, Changi General Hospital, Singapore, Singapore; 5 Department of Cardiology, Changi General Hospital, Singapore, Singapore; Universita degli Studi di Milano, ITALY

## Abstract

**Background:**

Elevated Lipoprotein(a) [Lp(a)] is a genetic risk factor for cardiovascular diseases affecting 20% of the world’s population, with multiple published consensus statements that recommend testing and management strategies. However, elevated Lp(a) remains under-detected and under-treated worldwide. Our qualitative study explored the perspectives of cardiology healthcare professionals regarding the barriers and enablers for Lp(a) detection and management.

**Methods:**

Guided by Theoretical Domains Framework, we conducted 41 qualitative semi-structured one-on-one interviews in a cardiology department at a high-volume hospital in Singapore from October to December 2023. Healthcare professionals were purposively sampled across role and seniority to include doctors (specialists and interns), specialist nurses and dedicated pharmacists. Through an inductive process, we constructed qualitative codes followed by code-mapping to arrive at higher-order sub-categories, categories, and eventually themes.

**Results:**

Analysis revealed 4 themes: rationale for routine testing, barriers to testing and follow-up, enablers of testing and follow-up, and ideal system to enhance patient management. Critical barriers to Lp(a) testing included a perceived lack of guidance in testing and follow-up, and misperception that Lp(a)-mediated cardiovascular risk cannot be managed resulting in low confidence of healthcare professionals to detect and manage elevated Lp(a). Inadequate institutional support to alleviate workload and presumed patient aversion to testing further hindered Lp(a) testing. We identified enablers and strategies to testing and management of Lp(a), notably these were the need for hospital-wide adequate training and education, guidelines and risk management pathways applicable to local settings, integration of Lp(a) testing into existing clinical pathways for high-risk patients, and user-friendly decision aids for healthcare professionals.

**Conclusion:**

Effective education for healthcare professionals and optimised clinical workflows may help to address current knowledge gap and implementation barriers in the detection and management of elevated Lp(a) in hospital.

## 1. Introduction

Lipoprotein(a) [Lp(a)] is a pro-inflammatory, atherogenic and thrombogenic type of lipoprotein that when elevated is associated with increased risk of atherosclerotic cardiovascular diseases (ASCVD) [1,2]. Concerningly, elevated Lp(a) affects one-fifth of the global population as the most commonly inherited condition related to hypercholesterolemia [1,2]. As ASCVD is one of the major leading cause of mortality and morbidity affecting one-third of global population, the pressing concern for adequate diagnosis and treatment has led to clinical recommendations and implementation suggestions by lipid and cardiology consensus statements [2–6] as well as the recent Brussels International Declaration to promote integration of Lp(a) testing and management as a necessary action into routine clinical care [7]. Given the evidence supporting Lp(a)’s role in cardiovascular risk reduction, multiple lipid and cardiology guidelines since 2017 have recommended a simple non-fasting blood test for high-risk individuals to detect the condition of elevated Lp(a). Some consensus statements and guidelines further advocate that all adults in the general population should also be offered Lp(a) testing at least once in their lifetime [2–6]. Upon detection of severely elevated Lp(a), cascade testing of family members is also recommended [1–3,5,8–11].

However, despite evidence-based guidelines and expert recommendations, many countries have not adopted routine Lp(a) testing in clinical practice [12–15]. A retrospective study of 70 million individual health records from a US-based international research network revealed a testing rate of only 0.1% [16]. The practice of Lp(a) detection and result interpretation varies greatly even among specialists managing ASCVD. Some overlook testing entirely: a study in Singapore found Lp(a) testing was not conducted on hospitalised patients with ischaemic heart disease or myocardial infarction prior to 2021, whereas other studies in different countries have also reported severe under-testing of Lp(a) in patients at very high cardiovascular risk [14,15,17,18]. A follow-up study in 2024 reported almost half of cardiology and endocrinology specialists in Singapore never tested for Lp(a) previously, reflecting major awareness gaps, partially contributed by lack of local guidance [18]. This under-detection and consequently under-treatment of elevated Lp(a) is alarming given the growing burden of ASCVD, especially in Asia where stroke and ischaemic heart disease consistently remain leading causes of death and the cardiovascular mortality rate in the region is expected to double by 2050 [19,20].

The definition of elevated Lp(a) is biochemical but hitherto there is no agreed universal threshold criterion. The 2022 European Atherosclerosis Society (EAS) consensus defined Lp(a) above 125nmol/L (50 mg/dL) as elevated Lp(a), but other expert consensus groups uses different threshold levels (e.g.,90 nmol/L, 100 nmol/L) [2,8,9,11]. The recently published European consensus (ESC/EAS 2025) acknowledges Lp(a) above 105 nmol/L (50 mg/dL) to be elevated [21]. Current recommendations for Lp(a) management include intensifying control of low-density lipoprotein cholesterol (LDL-C), blood pressure, diabetes, and obesity among other modifiable cardiovascular risk factors [2,8,9,11]. LDL-C reduction involves lipid-lowering agents targeting proprotein convertase subtilisin/kexin type 9 (PCSK9) pathways (e.g., evolocumab, alirocumab, and inclisiran), which also lower Lp(a) by 20–30%. This reduction likely accounted for the additional decrease of cardiovascular events in patients with elevated Lp(a) observed in post-hoc analyses studies [22,23]. Lipoprotein apheresis studies and large-cohort Mendelian randomisation analysis suggested that significant reduction of Lp(a) may decrease cardiovascular risk, thus providing impetus for rigorous management to mitigate adverse ASCVD outcomes [24,25]. Targeted Lp(a)-lowering agents (e.g., pelacarsen, olpasiran, and muvalaplin) have demonstrated encouraging clinical efficacy in Phase 1 and 2 studies [26–28], although results from its cardiovascular outcome trials are still eagerly awaited [9].

Given Lp(a)’s heavily overlooked potential to inform treatment and improve ASCVD prognosis, there is an urgent need to identify key obstacles to its detection and management, particularly in an Asian context. We conducted a pre-implementation study on the perspectives of healthcare professionals (HCPs) in a cardiology department of a large hospital in metropolitan Singapore. Our study aimed to explore the barriers and enablers affecting routine detection and management of elevated Lp(a) in patients at high risk for ASCVD and thereby inform the design of implementation strategies.

## 2. Methods

### 2.1. Study design

Three researchers (WJL, JJT, EL) developed a qualitative semi-structured interview guide (S1 Table) informed by the Theoretical Domains Framework (TDF), a validated framework comprising 14 domains: knowledge, skills, social/professional role and identity, beliefs about capabilities, optimism, beliefs about consequences, reinforcement, intentions, goals, memory/attention/decision processes, environmental context and resources, social influences, emotion, and behavioural regulation [29]. These domains encompass barriers to behaviour change at an individual level and can be used to select or design strategies to address modifiable barriers [30]. Since Lp(a) testing and management is initiated by individual clinicians, TDF was selected to underpin our study.

### 2.2. Study setting and participants

The study was conducted in Singapore, a multicultural hub in the Asia-Pacific region with a diverse ethnic population including Chinese, Malay, Indian and other ethnicities. Citizens rely on a robust public healthcare network well regarded for its efficiency, supported by national initiatives such as Healthier SG with a focus on preventive care and patient empowerment [31]. Participants were recruited from Changi General Hospital (CGH), a 1000-bed tertiary hospital serving a densely populated and aging community of over one million people in east and northeast Singapore. Handling among the highest cardiovascular workload in Singapore, its cardiology department recorded approximately 1000 admissions annually under the acute myocardial infarction pathway, along with many acute coronary syndrome cases admitted to other departments due to non-cardiovascular comorbidities.

HCPs in the cardiology department at the time of the study were invited to participate on a voluntary basis without reimbursement. Clinicians of varying seniority, together with dedicated nurses and pharmacists trained in cardiology care were purposively sampled to gain a range of perspectives within the department. Recruitment started on 18/10/2023 and concluded on 10/12/2023 upon data saturation, defined as the absence of further diversity of views or no new data. All participants provided informed written consent prior to the interviews. This study was approved by the SingHealth Centralised Institutional Review Board (reference number 2023–2429).

### 2.3. Procedures

Two researchers (BLP, JY) conducted 40 one-on-one interviews in-person and one remotely over Zoom, from October to December 2023. Researchers had minimal contact with participants before and after the interview concluded. Audio recordings were transcribed verbatim using an adapted version of Jeffersonian Transcription Notation (S2 Table) for consistency. Field notes replaced transcript for one interview (C013) due to technical errors. Transcripts were de-identified prior to data analysis.

### 2.4. Data analysis

Transcripts underwent analysis from April to June 2024 using Microsoft Word (2016, version 16.0.5452.1001). We conducted inductive coding followed by code-mapping to cluster codes into higher level categories and to generate themes. We elected not to conduct deductive coding based on the TDF for two reasons: to avoid imposing a pre-determined coding framework on the data and to allow a broader set of codes, beyond the level of individual actors, to be generated. The usage of TDF as a deductive coding framework would have restricted codes to the framework’s domains focussed on personal motivations and capabilities [32].

**Fig 1:**
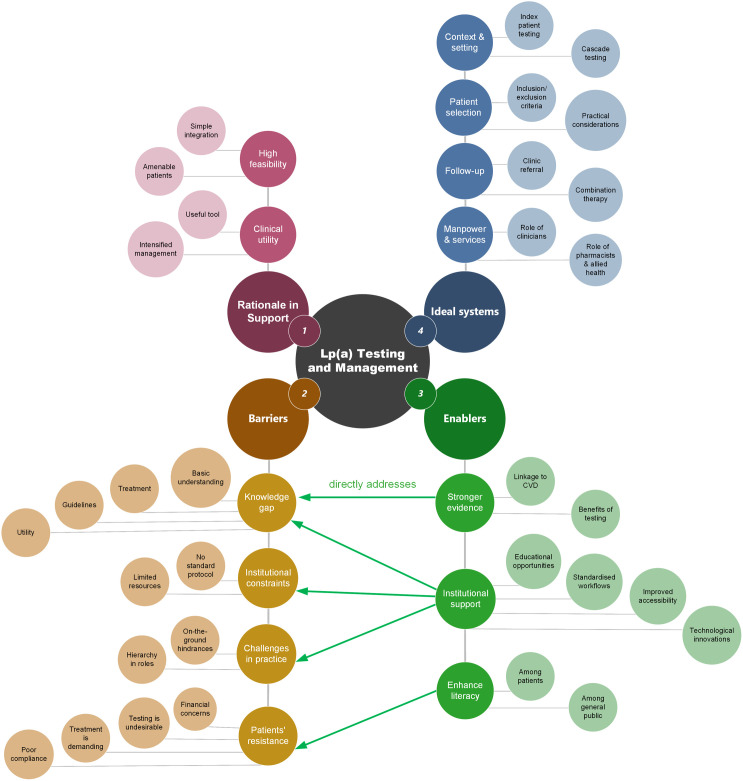
Thematic Network Diagram. Enablers were often discussed in direct response to the barriers, illustrated by arrows linking different categories.

Initially, four researchers (BLP, JY, LT, EL) read through the interview transcripts independently to familiarise with the dataset. The same three transcripts, randomly selected, were each examined independently by BLP, JY, and LT to identify ideas or concepts relevant to the research question from participant interviews. Each discrete idea or concept (a “unit of meaning”) was then given a label (a code). A codebook was developed iteratively as coding progressed. Codes were reviewed, and discrepancies were resolved via consensus discussion moderated by EL. The remaining transcripts were then randomly allocated to four researchers for independent coding (BLP, JY, LT, EL). Revised inductive codes were continuously reviewed as coding progressed (S3 Table). Two cycles of code-mapping were subsequently completed (LT, EL) to arrive at higher-order sub-categories, categories, and themes. The outputs were presented to the rest of the study team for sense-checking and refinement. We constructed a thematic network diagram to visually represent linkages between the themes and nested categories (Fig 1).

## 3. Results

The recruitment response rate was 80% (41/51). We interviewed 41 participants comprising 32 doctors (15 cardiology specialists, 5 cardiology registrars and 12 medical officers), 3 dedicated specialist nurses, and 6 dedicated pharmacists for an average of 15 minutes each. Table 1 shows the characteristics of participants and duration of interviews.

**Table 1:** Characteristics of participants (n = 41) and duration of interview.

Cardiology department role	Number of participants, n (%)	Response rate (%)	Male, n (%)	Years of experience, range (mean)	Interview duration, minutes (mean)
Consultant	15 (37)	94	73	7-22 (15)	7-38 (18)
Registrar	5 (12)	71	60	7-11 (9)	9-14 (11)
Medical Officer	12 (29)	75	33	1-15 (5)	4-22 (13)
Pharmacist	6 (15)	86	17	2-18 (10)	10-26 (18)
Dedicated Nurse	3 (7)	100	0	17-26 (21)	15-17 (16)

The process of inductive coding generated 106 codes, arranged into 13 categories and 32 sub-categories, and ultimately forming four themes regarding barriers and enablers of Lp(a) testing and management (S3 Table, Fig 1). To illustrate the different categories in each of the 4 themes, verbatim quotes from selected codes are provided in Tables 2–5, formatted in plain text without Jeffersonian Transcription Notation for ease of reading. Fig 1 illustrates the thematic network diagram to visually represent linkages between the 4 overarching themes and nested categories. Enablers were often discussed in direct response to the barriers, illustrated by arrows linking different categories.

**Table 2:** Categories, sub-categories and representative quotes under Theme 1.

	Code	Participants	Representative Quotes
***Category 1.1 High feasibility***			
1.1.1 Simple integration	Lp(a) testing is straightforward and can be easily incorporated into existing routine	Consultants, Medical officer (MO)	“When we educate the patients adequately the patients are – most probably they are ok, because this - this is not the invasive […] kind of blood test. For the admission patient we are doing the [other] blood test sometimes thrice a day sometimes once a day, so this is not – I think a big barrier for the patient.” (C024, MO)
1.1.2 Patients are amenable to testing	Lp(a) testing is deemed non-invasive		
	Patients are used to routine blood tests	MO	
***Category 1.2 Clinical utility***			
1.2.1 Useful tool in practice	Lp(a) is recommended by guidelines to be part of screening tool for routine ASCVD testing	Consultants, registrars	“We can show them that you know you have this marker that shows that your risk is high and these are all very well proven in the studies […] Abnormal Lp(a) result lends a lot of weight to helping us to maybe negotiate with the patients, especially those who are very resistant.” (C003, consultant)
	Lp(a) testing is useful as a risk stratification tool	Consultants	
1.2.2 Intensification of management	Abnormal Lp(a) result may lead to better patient management	Consultants, MO	

**Table 3:** Categories, sub-categories and representative quotes under Theme 2.

	Code	Participants	Representative Quotes
***Category 2.1 Knowledge gap among HCPs***			
2.1.1 Lack of understanding on Lp(a)	Association between Lp(a) and cardiovascular risk	Consultants, registrars, MO	“What I know is that the lipoprotein you know, increases your risk of IHD, but not sure about prevalence. […] I don’t think it’s part of the [ASCVD] risk calculator, but I assume that it probably does contribute to the actual risk.” (C023, MO)
	Awareness among clinicians and pharmacists of the importance of Lp(a)	Consultants, registrars, MO, pharmacists	
		Prevalence of high Lp(a)	
	Perception that Lp(a) is similar to LDL	Registrars, MO	
	Uncertainty over the role of Lp(a) in diagnosing familial hypercholesterolemia	MO	
2.1.2 Lack of effective treatment and evidence on patient outcome	Lack of treatment directly targeting Lp(a)	Consultants, registrars, MO	“Not that many widely available drugs that target Lp(a) specifically and even if there are, I think there is one, but I’m not sure about the clinical outcome benefits because it’s one thing to lower a number. It’s another thing to make sure that it produces outcome benefit.” (C032, consultant)
	Lack of outcome data demonstrating benefits of Lp(a) reduction	Consultant, registrar, pharmacists	
	Lp(a) treatment exists, but not approved for treatment in Singapore	MO	
2.1.3 Poor awareness of clinical recommendations	Lack of clinical knowledge regarding Lp(a) testing and treatment	Consultants, registrars, MO, pharmacists	“Consensus statements for testing Lp(a), the more contemporary ones were just released last year 2022. So I think perhaps a lot of people have not caught onto it […] even if they are aware, they might not be confident” (C030, consultant)
2.1.4 Limited utility of testing and treatment	Varying level of acceptance for Lp(a) testing as part of routine assessment	Consultants, registrars, MO	“If there is no cost effectiveness in screening because there is no way to treat it or even if you do know that you will have it, you can’t do much about it, then the test itself is very academic.” (C014, registrar)
	Elevated Lp(a) is not common enough to warrant routine testing	Consultants, MO	
	Lp(a) testing as part of routine screening may not result in meaningful changes to patient management		
	Potential medicolegal concern	MO	
***Category 2.2 Institution-level constraints***			
2.2.1 Lack of standardised management protocol	Lack of established routine to order Lp(a) testing	Consultants	“People may not have enough understanding of what this number actually means. If it’s high, what to do […] if it’s normal, it doesn’t mean that the patient is not at risk, right? […] GP may not be very familiar with Lp(a) and then you know if they just send the patients for like health screening and Lp(a) comes out, they may not know what to do with it. So then it will lead to a lot of referrals that may or may not be warranted.” (C016, consultant)
	Available guidelines on Lp(a) are unclear and do not offer enough direction		
	No Lp(a) target for follow-up	Registrars	
	Titrating treatment against changing guidelines is challenging	Pharmacists	
	Inconsistency in Lp(a) practices across individual clinicians	Consultants, registrars, MO	
	Prescribers’ comfort level with lipid-lowering therapy affect decision on combination therapy	Consultants	
	Institutional policies might be slow to adopt changes to Lp(a) management	Consultants, MO	
2.2.2 Limited manpower and resources	High attrition rate among clinical staff	Clinicians, nurses	“You get new doctors coming in every three months. And then how do you keep educating them to remember Lp(a)? Already have problems remembering some of the other more common things already how do you remember Lp(a)?” (C003, consultant)
	Concerns about manpower and workload to support follow-up after increased testing	Registrars, pharmacists	
	Patients with elevated Lp(a) requires counseling	Consultants	
	Potential issue managing patients in non-cardiology setting	MO	
	Difficulty testing patients in outpatient setting		
***Category 2.3 Challenges in practice***			
2.3.1 On-the-ground hindrances	Difficulties communicating implication of Lp(a) testing and management to patient	Consultants, MO	“It is not enough to tell the clinician please order Lp(a) because it never gets ordered, the MOs on the ground will be too busy running around to do other things.” (C003, consultant)
	Fast-paced working environment		
	Other works compete with Lp(a) screening	Consultants	
	Fatigue and stress		
2.3.2 Perceived clinical hierarchy influenced roles in clinical management	Workload and awareness of role are potential barriers for nurses to be involved in lipid management	Nurses	“I still feel the consultant […] should have more say, it is more of trying to sell the thing [treatment to the patient]. Like I say, it’s more of how our society is wired. The way that people think the doctor is always the expert already regardless; if it’s coming from them first, then subsequently when we [pharmacists] go in, anyone else go in, then it adds value. We are basically reiterating the point […] we are just a value-added partner.” (AH009, pharmacist)
	Doctors decide nurses’ involvement in educating patient about Lp(a) testing		
	Nurse reticence in reminding doctors to order Lp(a) test		
	Contentious whether to initiate PCSK9-targeted agents in outpatient or inpatient settings		
	Pharmacists struggle to convince patients to start PCSK9-targeted agents per doctor’s suggestion	Pharmacists	
	Prescribing etiquette for junior clinicians regarding combination therapy	MO	
***Category 2.4 Patients’ resistance to testing and treatment***			
2.4.1 Financial concerns	Concern on cost of testing	Consultants, registrars, MO	“If you’re telling me that, oh, this medicine is gonna save my life, but I have to empty out my bank to use it and have no more money to eat afterwards, who will do that? Right?” (AH004, pharmacist)
	Concern on cost of treatment	Consultants, MO, pharmacists	
	Potential implications on insurance coverage	Consultants, registrars	
	“You need to meet your basic needs first. Health usually takes a back seat”	Pharmacists	
2.4.2 Testing deemed undesirable	Testing might cause undue stress to patients	Consultants, MO	“You have to consider the impact of the patient […] This person is going to grow up knowing thinking that he’s going to get a heart attack, all the time, you know, the emotional duress.” (C031, consultant)
	Lack of motivation to test for a genetic condition with no treatment		
	Patients’ poor reception towards Lp(a) testing	Consultants	
2.4.3 Treatment deemed too demanding	Resistance to long-term treatment	Consultants, MO	“Most families and patients, one of their main concerns is trying to minimise too many visits to the hospital because normally either they’re working or the family is busy working […] it [PCSK9-targeted agents] is an injection if I recall right? They’re not very keen on injections. They prefer oral medications. ” (C027, MO)
	Reservation towards relatively unfamiliar treatment	MO	
	Injectable formulation is unpleasant	MO, pharmacists	
	Additional visits (if separate department is involved for follow-up) may discourage patients with time constraint from managing Lp(a)		
2.4.4 Poor compliance	Dosing schedule of PCSK9-targeted agents affects patient compliance	Pharmacists	“If you take something like once a week, twice a week, once a month, gets a bit odd. […] I had one patient who can’t remember, then he omit. […] if one guy has the problem, out in the community, say in a thousand people, how many will have the same problem as well?” (AH009, pharmacist)
	Conflicting advice from doctors regarding target lipid levels affects patient compliance		
	Poor medication compliance precludes therapy intensification	Consultants, MO	

**Table 4:** Categories, sub-categories and representative quotes under Theme 3.

	Code	Participants	Representative Quotes
***Category 3.1 Stronger clinical evidence base***			
3.1.1 Linkage to CVD	Strong evidence on the association of elevated Lp(a) with CVD risk can encourage testing	Consultants	“We should have our own data to convince the patient that measuring Lp(a) is important […] our own CGH database, whether it’s to show that it’s important to measure the Lp(a), to convince at least to convince the local- our CGH physicians […] that is important to measure Lp(a)” (C004, consultant)
3.1.2 Benefits of testing	Evidence showing Lp(a) testing will lead to better patient outcome can facilitate wider adoption of Lp(a) testing		
	Local data demonstrating benefits of Lp(a) measurement may lead to better uptake		
***Category 3.2 Institution-level efforts to support Lp(a) testing & management***			
3.2.1 Educational opportunities	Educational initiatives for HCPs	Consultants, MO, nurses	“Having some posters out in the on the lifts or waiting areas, then you know people can start reading and then they will be aware that this is something that is new or sending out emails, you know, promoting that we have this blood test and what is it for […] having some literature review, or journal club, during journal club sharing like for nurses or doctors even” (AH005, nurse)
	Education for clinicians on how to interpret Lp(a) results and what to do next	Pharmacists	
	Checklist of points for patient counselling on PCSK9-targeted agents		
	Preparedness of nurses to field questions from patients about Lp(a) testing	Nurses	
3.2.2 Standardised workflow	Standardised pathway for testing may encourage testing in practice	Consultants, registrars, MO, pharmacists	“At least from my experience with not just Lp(a) but with regards to everything else, very often pathways are very important […] once something is introduced into a pathway and then it gets ingrained as a culture, amongst the junior staff or all that, then it tends to be a automatic thing already […] build up awareness, put it into the pathways and then it will start – it will be automatic eventually” (C030, consultant)
	Well established guideline can improve management of elevated Lp(a)	Consultants, MO	
	Case managers are natural partners for patient education of Lp(a) testing	Nurses	
	Nurses can remind doctors to order Lp(a) testing if prompted by clinical pathway		
3.2.3 Accessible testing and treatment options	Subsidies for Lp(a) tests and treatment may improve detection rate	Registrars, MO	“But we do have our colleagues right, who work very closely with […] our telecarer, who calls the patient to reinforce and also our community nurse, our primary care partners […] we can’t see everybody in the whole community, but there’s the whole HealthySG, right, the initiatives and the intent. So we can probably build that components in our collaboration when we work with our primary care partners. So then they will help us to monitor them.” (AH006, nurse)
	Availability of treatment for high Lp(a) will increase the use of Lp(a) test		
	Improved access to testing and follow-up facilities may increase the use of Lp(a) test	MO	
	Partnership with healthcare providers in the community for long-term care post-acute myocardial infarction (AMI)	Nurses	
	Increasing Lp(a) testing and management needs a ministry-level top-down approach	Pharmacists	
3.2.4 Technological innovations	Improvement to current system interface may facilitate Lp(a) testing	Consultants	“[In hospital medical record system] Lp(a) is always forever in black, right? So you will never know whether it is high or normal […] if it’s more than 50, then it should be red. So people will know that it is a positive risk enhancer” (C016, consultant)
	Technology enhancement for patient care		
	Usage of certain Lp(a) assays		
	Inclusion of Lp(a) test into existing panel screening	Consultants, MO	
***Category 3.3 Efforts to enhance literacy among non-HCPs***			
3.3.1 Among patients	Patient education efforts	Consultants, pharmacists	“We empower the patient by giving the patient the information, lipoprotein (a) is high, what does this mean? If you are not sure, watch this video. If you want to do something about it, these are some of the options available” (C008, consultant)
	Explanation about Lp(a) level on health report can help with patient understanding	Consultants	
3.3.2 Among general population	General public awareness of the importance of Lp(a) testing	Consultants, MO	“We do educate patients regarding the importance of this and then of course from the policy side as well, where, you know, I think in alignment with a lot of our current policies to improve medical literacy. In alignment with you know HealthierSG policy and all that, so that will be the next stage” (C021, MO)
	Health literacy can be improved among general population to raise awareness of Lp(a)	MO	
	Role of allied HCPs in general	Consultants, MO, nurses	
	Role of dietitians	Consultants	

**Table 5:** Categories, sub-categories and representative quotes under Theme 4.

	Code	Participants	Representative Quotes
***Category 4.1 Context and setting of Lp(a) testing***			
4.1.1 Index patient testing	Appropriate setting to test for Lp(a)	Consultants, registrars, MO	“There’s very little benefit in doing more than once because Lp(a) is not supposed to fluctuate much during you know an individual’s lifetime […] there’s no proven therapy to significantly lower Lp(a) level as well. So little justification to have a repeat” (C002, consultant)
	Timing of Lp(a) testing is not critical	Consultants, MO	
	Repeat Lp(a) testing is not necessary		
	Possible use case for repeated Lp(a) testing	Consultants	
4.1.2 Cascade testing	Uncertainty about cascade testing for high Lp(a) levels	Consultants, MO	“If this 120 or even lower level has already resulted in a cardiac event like I said, you know very clean AMI, very clean you know ischemic heart disease and yet has borderline or, you know, highly elevated Lp(a) they will ask them to check because right now in cardiology we don’t have a cascade screening programme” (C031, consultant)
	Lack of recommendations and guidelines on cascade testing	Consultants	
	Possible use case for cascade Lp(a) testing		
***Category 4.2 Selection of patients for Lp(a) testing***			
4.2.1 Inclusion/ exclusion criteria	Appropriate patients for Lp(a) testing	Consultants, registrars, MO	“If someone is already like 80s or 90s and especially if they are just for medical therapy not for like procedure […] balancing the quality of life against doing more test and doing further evaluation and starting more intensive treatments, some of the patients, I would say not everyone would – might benefit” (C029, MO)
	Patients that do not need Lp(a) testing		
4.2.2 Practical considerations on Lp(a) testing	Patients eligible for Lp(a) testing, but at risk of not getting captured by current practice	Consultants	“Lp(a) can be done before discharge […] Not every patient will come for the post AMI clinic. […] maybe they are going to travel back to their own countries, patients who are going to go to another cluster because they live across the island. So we will miss out on a group of patients if we do it in outpatient” (C003, consultant)
	Pragmatic selection of appropriate patients for Lp(a) testing	Consultants, Registrars	
	Testing Lp(a) in patients with high LDL-C may improve high Lp(a) detection rate	Consultants, MO	
	Testing Lp(a) among patients in vascular department may improve high Lp(a) detection rate	Consultants	
***Category 4.3 Post-Lp(a) testing follow-up***			
4.3.1 Clinic referral	Management strategies/clinical actions following Lp(a) testing	Consultants, registrars, MO	“On top of – I mean the conventional treatments I guess also the role of other things like Repatha all this that can be added on top for these patients that are at a higher risk, especially with this genetic related […] it would be more intensive lipid lowering therapy and other adjuncts as well” (C029, MO)
	Criteria for referral to lipid clinic	Consultants, MO	
	Elevated Lp(a) threshold for referral to lipid clinic		
4.3.2 Combination therapy	Scenarios to start combination lipid lowering therapy	Consultants, registrars, MO	“LDL goal would be dependent on the risk of the patient. So for example, if they are intermediate and their Lp(a) is high, then they will be re-stratified to intermediate-high risk or even high risk and then their LDL goals will be adjusted accordingly. So it does affect the LDL goal” (C006, registrar)
	Opinion on LDL-C targets in relation to Lp(a) level		
	Lower LDL-C targets for patients with high Lp(a)		
	Specific LDL-C targets for patients		
	Understanding the barriers from patient’s perspective about PCSK9-targeted agents	Pharmacists	
***Category 4.4 Manpower/ Services to support Lp(a) management***			
4.4.1 Role of clinicians at different levels	Seniority level of clinicians required to follow up and manage high Lp(a)	Consultants, registrars, MO	“If we are going to look at Lp(a) as a - as a prognostic marker for all primary prevention in terms of cardiovascular and health, then I think optimally and ideally all primary care physicians should be equipped to managing it. […] in the long run what we should really be looking at is everyone being equipped to deal with it. And any seniority level. Like anyone who’s running a clinic in the polyclinic or even in the GP setting, they should be able to deal with it” (C032, consultant)
	Specialty to follow-up and manage high Lp(a)		
	Healthcare services to follow up and manage Lp(a)		
4.4.2 Role of pharmacists and allied health professionals	Role of pharmacists	Consultants, MO, pharmacists	“I think it should be a joint force. I mean we work as a team as well. […] so that everybody can just start reminding the patient, say why is it important and why we actually recommend this so that everybody is on the same page […]. So a joint team effort would be a better option definitely.” (AH005, nurse)
	Role of allied HCPs in general	Consultants, MO, nurses	
	Role of dietitians	Consultants	

### 3.1. Theme 1: Rationale for routine testing

Theme 1 (Table 2) captures participants’ rationale for Lp(a) routine testing in hospitals. First, testing was generally perceived to be straightforward and feasible, easily done on-site, and is not more invasive than routine blood tests. Second, the clinical utility of Lp(a) testing was appreciated as part of prevention based on recommendations from existing guidelines. However paradoxically, we noted low awareness of guidelines on target population and management strategies. Third, Lp(a) testing was perceived as useful for patient stratification, thus empowering HCPs to intensify treatment and reducing clinical inertia. Similarly, patients might be more open to aggressive lipid management strategies given an elevated Lp(a) result.

### 3.2. Theme 2: Barriers to testing and follow-up

In contrast to Theme 1’s perceived advantages of Lp(a) testing, Theme 2 (Table 3) explores current challenges and foreseeable barriers leading to scepticism towards the diagnosis and management of elevated Lp(a) in practice.

#### 3.2a). Knowledge gap among HCPs.

The first and foremost barrier was a lack of medical knowledge. Regarding general understanding of Lp(a), almost all reported uncertainty on pathophysiology, prevalence and risk thresholds; including clinicians who acknowledged elevated Lp(a) as a risk factor for ASCVD. The clinical significance of Lp(a) in ASCVD management was deemed unestablished: Lp(a)’s role as a risk factor only gained prominence recently and key strategies such as cascade testing was unknown to many. Given the recency of consensus on management strategies (within the last 5–7 years), HCPs especially non-cardiologists or non-endocrinologists were likely not abreast regarding recent developments on testing and treatment. This perceived lack of clinical recommendations on both patient selection and subsequent follow-up emerged as a major barrier to routine testing.

Beyond limited knowledge leading to poor capability to manage patients, Lp(a) testing and treatment was also deemed futile. Elevated Lp(a) was considered relatively uncommon, casting doubt over cost-effectiveness of routine testing. Another common perception was that patients’ current lipid-lowering regime is already optimised with little room for more intensive intervention. Therefore, even with increased diagnosis, existing patient management strategies would likely remain unchanged.

There was also a perception that no effective Lp(a) treatment currently exists to warrant testing, as typical lipid-lowering medications yielded insufficient Lp(a) reduction. Targeted agents such as muvalaplin were still undergoing clinical trials. Other interventions such as plasma apheresis were locally unavailable as a therapeutic option and its cost-effectiveness untested. Lifestyle modifications such as exercise and diet management were deemed ineffective. Equally vexing was the perceived lack of conclusive evidence that reducing Lp(a) improves patient outcomes. Consequently, there was concern that Lp(a) testing might present medico-legal and insurance claims issues, as diagnosing an inheritable condition without effective follow-up strategies would raise the spectre of medical negligence.

#### 3.2b). Institution-level constraints.

Another key barrier was institution-level roadblocks, particularly limited formalised local guidance, which together with the knowledge gap, further contributed to reticence towards Lp(a) testing. Self-reported differences in practice ranged from ordering Lp(a) tests once or twice to testing every patient. This inconsistency was attributed to differing emphasis on Lp(a) management and unclear guidelines. Guidelines and actual practice being in flux complicated treatment decisions: pharmacists reported difficulty titrating medications against constantly changing targets in lipid management without a clear timeline for therapy and lipid targets. Embedding standard practices typically takes time due to the slow pace of adopting new guidelines in large institutions, further aggravating the issue.

Current manpower resources were also deemed insufficient to support Lp(a) management, especially in non-cardiology settings. High turnover of staff necessitates constant re-training of incoming junior HCPs on testing procedure, counselling approaches, and long-term management, which may stretch beyond the index case to family members for cascade testing.

#### 3.2c). Challenges in practice.

The already thin workforce is further strained by daily on-the-ground realities such as fatigue. Difficulty communicating with patients in a stressful working environment, coupled with multiple competing concerns to attend to, further burdened HCPs.

Clinicians reported logistical roadblocks in outpatient clinic setting such as difficulty capturing potential patients within the appointment period, significant time lag between ordering the test and receiving results, restrictions in prescribing privileges or referral to the relevant specialists or clinics for follow-up. These challenges informed a preference for inpatient management and the belief that the responsibility for detecting and managing elevated Lp(a) patients largely rested with the inpatient team.

Nurses and pharmacists offered a different view: while intervention might be initiated in the inpatient setting, outpatient clinics should be responsible for long-term follow-up. Contrary to junior doctors who reported no issues raising concerns on treatment strategies to their superiors, nurses and pharmacists were reluctant to do so, preferring instead to be delegated tasks and to reiterate clinicians’ recommendations. Pharmacists doubted their role and capability to counsel patients to accept treatment, citing patients’ deference to doctors. The perceived lower clinical influence compared to clinicians suggested a hierarchy in clinical decision-making among HCPs, further hindering pharmacists and nurses from proactively engaging in Lp(a) management.

#### 3.2d). Patients’ resistance to testing and treatment.

Tangential to on-the-ground constraints for HCPs were patient-related challenges; specifically, a presumption that some patients might be resistant to Lp(a) testing and treatment. Financial concern was presumed a key issue since testing may incur out-of-pocket expenses for the patient. However, many participants could not provide cost estimates for the test and recanted their opinion when informed that the actual cost of Lp(a) test was lower than SGD $40 (USD $30) per test for patients in our hospital. The cost may vary depending on patient’s subsidy program, with the highest rate of SGD $60–70 (USD $50) for patients in private class. Treatment cost was also a potential barrier, with reports of patient complaints or treatment refusal, citing long-term financial burden as a key reason. Furthermore, there was concern that an elevated Lp(a) result might hypothetically affect patients’ future eligibility for health insurance, leading to higher insurance premiums and restricted access to affordable care.

Apart from financial concerns, patients might be unmotivated to test and confirm the diagnosis, given the condition’s genetic underpinning with no apparent cure and the emotional distress that might follow. Long-term treatment might be considered demanding and inconvenient, especially self-injectable PCSK9 inhibitors or newer Lp(a) lowering agents. The use of needles for administration, dosing schedule that might require recurring visits to specialists, and relative unfamiliarity compared to oral medications such as statins, might contribute to treatment aversion. Patient adherence to treatment was also a concern. Adding or intensifying existing medications to target Lp(a) or overall lipid control, especially given the lack of clear unified guidance on management might risk treatment failure.

### 3.3. Theme 3: Enablers of testing and follow-up

In response to the barriers in Theme 2, Theme 3 (Table 4) discussed enablers of Lp(a) testing and management.

#### 3.3a). Stronger clinical evidence.

To address the knowledge gap barrier, stronger clinical evidence may convince HCPs that elevated Lp(a) warrants treatment. Specifically, data that clearly demonstrates improvement in clinical outcomes among the local population following testing and prescribing of Lp(a)-lowering agents.

#### 3.3b). Institution-level enablers.

Many of the barriers identified such as poor awareness of Lp(a), lack of standardised protocol, limited resources and on-the-ground challenges in practice stem from underlying systemic constraints. Participants felt that a multi-pronged top-down approach, initiated by healthcare authorities and providers, is needed to effectively incorporate Lp(a) into mainstream care practices.

Within the healthcare institution, educational initiatives can boost awareness of Lp(a)’s role in ASCVD, especially among non-cardiologists, nurses, and pharmacists. Continuing medical education is needed on test result interpretation and management strategies. For pharmacists, a checklist for patient counselling on treatments such as PCSK9-targeted agents would be useful. Standardised workflows and clinical protocols should be established for Lp(a) testing with clear patient inclusion criteria; and integrated into existing protocols where appropriate. For instance, the Lp(a) test should be included in existing blood panels for ease of ordering [33]. Patient-facing materials such as health reports could provide additional information about Lp(a) in plain English.

Technological innovations may further enhance Lp(a) management in practice. Existing medical record systems can be improved to support clinical decision-making: electronic reminders prompting clinicians to order Lp(a) test for suitable patients upon admission; pre-programmed clinical checkpoints highlighting missing result or skipped test; and clear visual displays to aid result interpretation based on pre-programmed Lp(a) thresholds requiring clinical action. Social media and mobile applications (health apps) can be harnessed for patient education to encourage a healthy lifestyle between clinic visits.

Improved treatment affordability and subsidies may lower financial barriers and encourage HCPs and patients to proceed with Lp(a) testing. However, funding would require involvement of the local ministry of health. A national cross-sector approach may support long-term management, including national clinical protocols, and provider partnerships beyond hospitals and clinics.

#### 3.3c). Enhance health literacy.

In addressing the perceived patient resistance against testing and treatment, healthcare institutions could engage community outreach activities to improve awareness on Lp(a), its health implications, and encourage testing among the general population. Suggestions included leveraging the institution’s social media presence via posts or blogs and partnering with relevant health promotion agencies, thus improving health literacy for the general population.

### 3.4. Theme 4: Ideal system to enhance patient management

**Fig 2:**
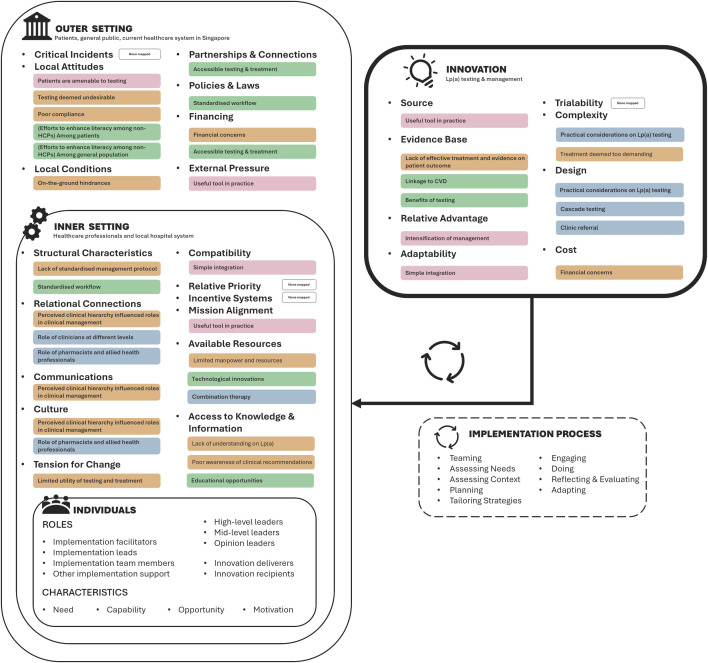
Mapping of 32 sub-categories from the study to 26 CFIR constructs in the Innovation, Outer Setting, and Inner Setting domains.

Theme 4 (Table 5) describes participants’ perception of what Lp(a) testing and management should entail, given the constraints and possible solutions they proposed in Theme 2 and 3, respectively. Fig 2 shows the mapping of the 32 sub-categories from the study to 26 Consolidated Framework for Implementation Research (CFIR) constructs in the Innovation, Outer Setting, and Inner Setting domains.

#### 3.4a). Context and setting for Lp(a) testing.

Inpatient was the ideal opportunistic setting to initiate testing and follow-up before discharge, especially in cardiology and vascular departments. Selected outpatient clinics might also initiate testing, for example post-acute myocardial infarction clinics. Clinicians reported no preferred testing period following an index cardiovascular event. Repeat testing was deemed unnecessary unless the result was ambiguous or for monitoring response to treatment. Clinicians were in favour of cascade testing beyond the index case when provided with additional explanation.

#### 3.4b). Selection of patients for Lp(a) testing.

Inclusion criteria varied significantly between all cardiology inpatients (including non-ASCVD cases such as atrial fibrillation) and favoured a targeted selection to conserve limited resources. Specifically, a sub-population of ASCVD patients should be prioritised: those without apparent or well-controlled cardiovascular risk factors, younger people (40–50 years old), with history of familial hypercholesterolaemia or referred for cascade testing. Conversely, there were also opinions by cardiologists that older patients (>80 years old), those with lower risk profiles (non-cardiac related symptoms, low cholesterol level, stable condition) were deemed unlikely to benefit and should be excluded.

#### 3.4c). Post-Lp(a) testing follow-up.

Participants reported different, at times contradicting, viewpoints on treatment strategies. While clinicians agree that lipid-lowering regime can be strengthened by either increasing the dosage of existing medications or adding PCSK9-targeted agents as part of combination therapy, opinions differed regarding lifestyle modifications as useful adjuncts for a genetically determined condition. Management strategies included referral to the hospital’s lipid clinic, albeit without a consensus on Lp(a) referral thresholds ranging from 60 to 150 nmol/L. Uncertainty about LDL treatment goal post-diagnosis was also reported.

#### 3.4d). Resources to support Lp(a) management.

Lp(a) management should be a cross-specialty team effort preferably led by experienced cardiologists or endocrinologists (lipid specialists). Having multi-disciplinary HCPs involved in an integrated clinic would promote collaborations and optimise patient care. Specialist pharmacists and allied health professionals could play a proactive role: pharmacists are well-positioned to address medication adherence issues, while dietitians could provide dietary education and strategies for healthy eating. With sufficient training, other clinicians including general practitioners can be in charge. Over time, elevated Lp(a) should ideally be managed in the primary healthcare setting.

## 4. Discussion

Our study captured new insights and reaffirmed previously known barriers and enablers for Lp(a) testing and management. First, low awareness of Lp(a) even among specialists remained a major and well reported hurdle that contributed to low adoption of Lp(a) testing [15,17,18,34]. Misperceptions such as futility of testing, low utility in CVD risk re-classification, perceived lack of actionable guidelines and effective therapeutic options contradicted current evidence [2–5,8,9]. These misperceptions were prevalent despite extensive coverage about Lp(a) in conferences, medical resources, and international guidelines published between 2018 and 2023 prior to our study. Therefore, the need remains for extensive continuing medical education to fill knowledge gaps and address misconceptions, and for the development of local protocols based on international guidelines and consensus [2]. The participants of this study voiced their opinions that more studies are needed on the clinical significance of Lp(a) in the local and broader Asian setting to better support its implementation as an important risk factor into practice, not just to reflect current understanding of the topic based on international guidelines but also to contribute towards Healthier SG’s goal on preventive care in an aging population [31].

Second, participants cited unaffordability of testing and treatment as a key patient deterrent. Interestingly, this concern stemmed from a perceived notion that Lp(a) testing was too expensive rather than knowledge of the actual cost. Interestingly, many participants changed their opinion to be now in favour of testing, when informed of the actual cost of Lp(a) testing which was cheaper than renal, liver or thyroid panel in our hospital. Therefore, this perceived barrier of ‘expensive test’ can easily be rectified with adequate information dissemination including explanation that most patients only needed to be tested once if normal. While the solutions to patient’s financial issues lie beyond individual clinicians’ capability, more clarification on the cost and subsidies on testing and treatment would help HCPs be better informed to counsel patients accordingly. High-risk patients were also thought to be resistant to testing and treatment procedures, although patient advocacy groups would likely disagree with such a presumption. A high level of public awareness is a critical enabler for large-scale Lp(a) testing and management, and implementation efforts are necessary to improve health literacy among patients, families and public.

Third, junior clinicians, nurses and pharmacists also expressed low confidence and were forthright about their lack of experience and understanding when discussing Lp(a) testing and management. While the test can be conducted fairly easily, there was an unsubstantiated perception that Lp(a) detection and by extension its management exceeded the current capabilities of junior HCPs, although participants also expressed an openness to rectify their limited understanding. Suggested educational modalities included topic presentations of Lp(a), workshops, case-based discussions, journal clubs, internal emails, posters, and brochures: focussing on knowledge of aetiology and prevalence, testing instruction, result interpretation, counselling points, and other patient management skills. Notably, there was a heartening sense of eagerness among participants to be well-prepared, so any HCP involved in the ASCVD care pathway can competently address questions from patients about Lp(a) testing and management. Given the high degree of overlap between elevated Lp(a) and high ASCVD risk, Lp(a) training efforts should also be extended beyond cardiology HCPs to include medical officers, interns, nurses and allied health at acute admission wards.

Key insights from this study have enabled implementation of Lp(a) into patient care in our hospital locally. Efforts consisted of an Lp(a) guide for healthcare professionals made available on the hospital’s intranet and an easy referral system to the Lipid Clinic for management of patients with elevated Lp(a). An Lp(a)-cardiology pathway was launched, accompanied by a series of educational talks on Lp(a) using the *#LILAC-for-Lp(a)* concept to HCPs [35]. As ASCVD management is multidisciplinary, the educational talks on Lp(a) were not limited to cardiology and endocrinology departments, but also included internal medicine, stroke, rehabilitation, surgical departments, allied health, nursing, and primary care. Other implementation efforts included Lp(a) educational awareness events, notably Singapore’s inaugural Lp(a) Awareness Day event for HCPs, patients and public held in CGH on 23/04/2024, followed by a ‘Changi General Hospital Lipid and Lp(a) Awareness Week’ in April 2025 [35,36]. From our experience, the use of the novel *#LILAC-for-Lp(a)* concept via a short 8-minute educational video was particularly useful to positively influence practicing doctors, nurses and allied health professionals to test and manage Lp(a) [35]. The LILAC mnemonic was designed by the corresponding author (WJL) as a cognitive-aid tool to remind HCPs to use Lp(a) to improve cardiovascular risk stratification, and importantly, to advocate a healthy lifestyle and control other cardiovascular risk factors such as elevated LDL-C, blood pressure, blood glucose, and obesity [35].

With the implementation of these strategies informed by our study, Lp(a) testing has improved significantly to a sustained rate of >80% of all patients admitted to our hospital for acute myocardial infarction [37]. More than 2000 cardiology patients with high cardiovascular risk were tested for Lp(a) one year after integration of Lp(a) into cardiology pathways, of which 16% of patients were detected to have elevated Lp(a) [37]. Our positive experience of integrating Lp(a) into clinical care is in line with other centre’s experience that integration of Lp(a) into lipid profiling in Lipid Clinic and clinical care helps improves cardiovascular risk stratification of patients at high cardiovascular risk, including patients with familial hypercholesterolaemia and/or have ASCVD [38,39].

Such encouraging outcomes of local implementation efforts demonstrate that the pre-implementation findings reported in this study can be translated into impactful strategies, benefitting not just patients and individual HCPs in practice but also the broader healthcare community and institution. While being guided by an individual-centric framework (the TDF) for data collection, our approach to data analysis using inductive coding yielded insights that influence implementation on a wider scale, beyond behavioural change at an individual level. In particular, the findings are concordant with the domains covering intervention characteristics, institutional setting, and wider societal context as articulated in the Consolidated Framework for Implementation Research (CFIR) [40]. Fig 2 demonstrates how each construct under these CFIR domains can be mapped one-to-many to the sub-categories generated in our study. Future research may choose to use the CFIR framework to focus on constructs not extensively covered in our study such as trialability, critical incidents, relative priority and incentive systems for Lp(a) testing and management. In view of the recent implementation efforts as described, subsequent studies may further use CFIR’s individuals and implementation process domain as guidance to identify relevant stakeholders, post-implementation challenges and follow-up strategies to effectively support diagnosis and treatment.

This study has several limitations. First, recruitment was limited to HCPs in the cardiology department at a single large healthcare institution. However, we recruited clinicians of various seniority, nurses, and pharmacists, many of whom rotate across multiple similarly resourced hospitals during training in a culturally diverse community. Thus, we believe their views would be broadly consistent with peers in other institutions and generalisable to a certain extent across the Asian context. We did not interview patients, hence the views offered by HCPs on patients’ preference are only presumed. Strengths of this study include a theory-driven qualitative approach and a multidisciplinary study team comprising an endocrinologist (WJL), a cardiologist (JJT), pharmacists (BLP, JY), and researchers in health services research and implementation science (EL, LT).

## 5. Conclusion

This pre-implementation study identified critical barriers to be addressed at the individual and institutional level to optimise adoption of Lp(a) testing and management. Foremost of these barriers is the major knowledge gaps regarding the importance, utility, and benefits of Lp(a) testing and its subsequent management amongst HCPs and patients. Effective education strategies and protocol-driven clinical workflows are needed to increase Lp(a) testing and management. The enabling strategies proposed by the participants corresponded to key barriers identified and thus informs future implementation.

## Supporting information

S1 Table Interview Guide. (PDF)

S2 Table Adapted Jeffersonian Transcription Notation. (PDF)

S3 Table Codebook. (PDF)
