# Supplementary material for: Barriers and strategies in detection and management of elevated Lipoprotein(a) in hospital: A pre-implementation qualitative study of cardiology healthcare professionals
Source: PLoS One. 2025 Oct 15;20(10):e0333789. doi: 10.1371/journal.pone.0333789 (PMC12527205; doi:10.1371/journal.pone.0333789)
Supplement: S1 Table — (PDF) [file pone.0333789.s001.pdf]

Table S1. Interview Guide

No.	Questions and Follow-up Prompts
**Cardiology Consultants**	
1	What is your opinion of measuring Lp(a) routinely in your clinical practice for patients with AMI/IHD in both inpatient/outpatient setting?<br>- Why would you measure/not measure?
2	Who do you think should be following up/managing patients with high Lp(a)?<br>- Which discipline and what seniority level of doctors?
3	What is your threshold for referring patient with high Lp(a)/LDL/triglycerides to lipid clinic?
4	What do you think are the potential barriers in screening and managing patients with high Lp(a)?
5	Who and when do you think should we do cascade testing for high Lp(a)?
6	What do you think are the allied healthcare professionals' roles (e.g. pharmacist, dietitian) in lipid management?<br>- Apart from those that they are currently doing?
7	In your opinion, should Lp(a) measurement be a routine part of cardiovascular risk assessment?<br>- Why/why not?
**Cardiology Registrars and Medical Officers**	
1	What do you know about Lp(a) and its prevalence?
2	How do you think we can improve the detection rate for high Lp(a)/Familial hypercholesterolemia?
3	What are the challenges you think you may face when screening/managing patients with high Lp(a)?
4	Who do you think should be following up/managing patients with high Lp(a)?<br>- Which discipline and what seniority level of doctors?
5	In your opinion, should Lp(a) measurement be a routine part of cardiovascular risk assessment?<br>- Why/why not?
6	What is your opinion of prescribing combination therapy (statin/ezetimibe/PCSK9-targeted agent) in lipid management?
**Dedicated Nurses and Pharmacists**	
1	How do you think pharmacist or nurse can play a part in lipid management e.g. high Lp(a)?
2	Would you be agreeable to the idea of pharmacist or nurse doing counselling to eligible patients on starting PCSK9-targeted agent?<br>- If not, who do you think should be responsible?
3	If we were to implement pharmacist or nurse counselling for PCSK9-targeted agents initiation, how do you think we can do this?
4	How do you think we can improve the awareness of Lp(a) screening and its management?
5	What do you think are the benefits of involving pharmacist or nurse in the management of high Lp(a)?
6	What do you think are the potential barriers that pharmacist or nurse will face while playing a part in lipid management?
