# Supplementary material for: Barriers and strategies in detection and management of elevated Lipoprotein(a) in hospital: A pre-implementation qualitative study of cardiology healthcare professionals
Source: PLoS One. 2025 Oct 15;20(10):e0333789. doi: 10.1371/journal.pone.0333789 (PMC12527205; doi:10.1371/journal.pone.0333789)
Supplement: S2 Table — (PDF) [file pone.0333789.s002.pdf]

Table S2. Adapted Jeffersonian Transcription Notation

Symbol	Usage
[text]	Indicates text inserted by researcher. For example [participant was interrupted by their mobile phone ringing]
(number of seconds paused)	Indicates the approximate time in seconds of a pause in speech. For example, if the participant paused for 2 seconds, annotate as (2)
-	Indicates an abrupt halt or self-interruption while speaking. For example, when the participant is answering, they cut themselves off: "You know, it's really- I mean it's really hard to tell sometimes."
=	Indicates the break and subsequent continuation of a sentence. For example:<br>Researcher: That's interesting, could=<br>Participant: What?<br>Researcher: =could you tell me more about that?
_____	Underlined text indicates the participant is emphasizing or stressing the word/s. For example: "I would love to see that."
:::	Indicates prolongation of a sound/a "stretched" sound. For example: "Ye:::ah, maybe"
(text)	Words that are unclear or in doubt in the transcript. For example: "I don't know what's the (problem?) with the group. Sometimes, it's (inaudible) and other times they're OK."
((text))	Annotation of non-verbal activity. For example: "Well, I think it's OK ((laughs)). As long as it doesn't affect anyone else? ((looks unsure)).
