# Supplementary material for: Barriers and strategies in detection and management of elevated Lipoprotein(a) in hospital: A pre-implementation qualitative study of cardiology healthcare professionals
Source: PLoS One. 2025 Oct 15;20(10):e0333789. doi: 10.1371/journal.pone.0333789 (PMC12527205; doi:10.1371/journal.pone.0333789)
Supplement: S3 Table — (PDF) [file pone.0333789.s003.pdf]

Table S3. Codebook

Theme	Category	Sub-category	Code	Description	Representative Example
Rationale for Routine Testing	High feasibility	Simple integration	Lp(a) testing is straightforward and can be easily incorporated into existing routine	Participants believe that initiating Lp(a) testing is simple and should not pose a challenge to current procedure	C025: Participant identified no challenges as ordering Lp(a) bloodwork is straightforward "a simple blood test to be added onto normal lipid panel".
		Patients are amenable to testing	Lp(a) testing is deemed non-invasive	Most patients perceive Lp(a) testing as non-invasive and are thus open to be tested	C027: Patients are generally okay with Lp(a) testing since it does not require additional venipuncture if patient is already being screened
			Patients are used to routine blood tests	Inpatients might already be familiar with frequent blood tests and are thus not resistant to Lp(a) testing procedure	C024: Admission patients sometimes undergo up to 3 blood tests per day, so Lp(a) testing does not present a big barrier
	Clinical utility	Useful tool in practice	Lp(a) is recommended by guidelines to be part of screening tool for routine ASCVD testing	Participants believe that Lp(a) should be added into routine ASCVD screening as per guideline	C032: Lp(a) testing is recommended in cardiology guidelines to be done as screening tool for primary prevention
			Lp(a) testing is useful as a risk stratification tool	Elevated Lp(a) is a risk factor for CVD and testing for Lp(a) can be a way to stratify patients based on risk profile	C002: Lp(a) is a simple test to help stratify patients' risk level to decide how aggressively to manage them, whether to start statins for treatment
		Intensification of management	Abnormal Lp(a) result may lead to better patient management	Participants share the different ways patients might be more open to management strategies and clinicians might be empowered to push for more aggressive LDL control (avoiding clinical inertia)	C003: Benefits outweigh cost – information on abnormal Lp(a) level might be useful to convince patients to achieve lower LDL target, especially for those resistant to medication change.
Barriers to Testing and Follow-up	Knowledge gap among HCPs	Lack of understanding on Lp(a)	Association between Lp(a) and cardiovascular risk	Participant describes Lp(a) as one of the risk factors for cardiovascular diseases. High Lp(a) increases CV risk especially in younger patients	C022: Participant was uncertain, believed lipoprotein increases IHD risk, did not think it is part of the risk calculator but assumed it does contribute to the actual risk
			Awareness among physicians and pharmacists regarding importance of Lp(a)	Participants believe that not many know about the importance of Lp(a) currently.	C019: Lp(a) testing and subsequent management of it is quite new and only common in cardio/endocrine but not other departments
			Prevalence of high Lp(a)	Participants are uncertain about the	AH002, 003, 004, 007: all pharmacists (senior and

				prevalence of high Lp(a) in general population	junior) expressed uncertainty about the prevalence of high Lp(a) in the general population.
			Perception that Lp(a) is similar to LDL	Participant believes the role of Lp(a) is similar to that of LDL	C024: Participant believed Lp(a) is "a type of cholesterol" that causes atherosclerotic plaque formation "almost […] as equal as LDL" when asked to compare with other types of cholesterol
			Uncertainty over the role of Lp(a) in diagnosing familial hypercholesterolemia	Participant was uncertain of the association between Lp(a) and FH in context of diagnosis	C028: Participant was initially under the impression that Lp(a) is associated with familial hypercholesterolemia and that elevated Lp(a) is indicative of FH thus there is a chance of false positives in testing. Participant then clarified that both may occur in isolation.
		Lack of effective treatment and evidence on patient outcome	Lack of treatment directly targeting Lp(a)	Participants noted ongoing trials and uncertainties about how to manage high Lp(a) levels effectively	C014: No effective treatment – none is currently available or commercially available yet and hence there is nothing to do medically about elevated Lp(a). There may be incoming trials to find effective molecular target. Plasmapheresis may be able to treat the underlying condition, but is not very cost-effective as it is only applicable for a very selective group of patients and not available at every centre.
			Lack of outcome data demonstrating benefits of Lp(a) reduction	Participant highlights the absence of conclusive outcome data demonstrating that reducing Lp(a) levels leads to improved patient outcomes.	C032: Even if there are medications to lower Lp(a), there is no current evidence suggesting that lowering Lp(a) will improve patient outcomes "it's one thing to lower a number. It's another thing to make sure that it produces outcome benefit"
			Lp(a) treatment exists, but not approved for treatment in Singapore	Treatment options targeting Lp(a) are limited and not currently in practice locally	C024: Muvalaplin, a current option for Lp(a) control, is not prescribed in Singapore. Plasma apheresis is also another option that MO did not observe any patients undergoing for now
		Poor awareness of clinical recommendations	Lack of clinical knowledge regarding Lp(a) testing and treatment	Participants are unsure about how to manage patients, who should be screened for Lp(a), the interpretation of Lp(a) level, and its treatment<br><br>Pharmacist-Participants are unsure how Lp(a) levels affect lipid management.	C030: Clinicians might be aware of the importance of Lp(a), but are still reluctant to order testing as they are unsure about guidelines and not confident enough to follow up
		Limited utility of	Varying level of acceptance for	Participant expresses their personal	C021: Participant was cautious "based on my

		testing and treatment	Lp(a) testing as part of routine assessment	opinion on whether Lp(a) testing should be done as part of routine CVD risk assessment	understanding I would say yes but I don't have that much understanding", needs more information on pretest probability and risk profiling
			Elevated Lp(a) is not common enough to warrant routine testing	Participant believes that Lp(a) should not be added into routine ASCVD screening as it is relatively uncommon among patients, probability might not be high enough for cost-effective screening	C016: Testing should be on a case-by-case basis (small population, young patients with no family history) and not blanket testing. The latter might not be cost effective
			Lp(a) testing as part of routine screening may not result in meaningful changes to patient management	Participants share the different ways patient management might not change following Lp(a) testing	C016: Participant believed Lp(a) testing does not carry much clinical benefits beyond counselling patients for lifestyle modifications.
			Potential medicolegal concern	There may be future medicolegal issues should current patients are tested but not treated	C018: Participant was concerned that screening for Lp(a) when there is no effective treatment now may become a medicolegal issue if there are proven implications on cardiovascular risks but patient was not treated appropriately
	Institution-level constraints	Lack of standardised management protocol	Lack of established routine to order Lp(a) testing	Due to the novelty of Lp(a), testing procedure is not frequently put into practice enough for clinicians to remember ordering the test	C003: Clinicians are aware of the significance of Lp(a) testing but forget to check patient's records and order test if needed, as it is not part of an established work routine. People may understand the importance, but do not order the test in practice out of habit.
			Available guidelines on Lp(a) are unclear and do not offer enough direction	Current guidelines do not offer clear instructions and might not instill confidence in clinicians for treatment and follow-up	C032: Different cutoff thresholds offered by different guidelines: original ESC guideline uses 50 and 125, while latest guidelines give a higher cutoff for greater predictive value. Participant was unsure when asked about prevalence based on cutoff
			No Lp(a) target for follow-up	There is no Lp(a) target for patients to aim for as follow-up after testing	C006: Participant suggested that there are no target for Lp(a) because current therapy like statins do not actually lower Lp(a) and it is only a once off measurement
			Titrating treatment against changing guidelines is challenging	Titrating treatment against changing guidelines is challenging, and the lack of clear timelines for achieving [lipid] goals makes it difficult to judge when	AH004: "I think will be a clear direction of what targets to meet. Currently our lipid clinic's guidelines is based on the - I cannot remember, is it the Lipid Guideline 3 and lipid guideline 4. One ask

				to intensify therapy.	for target and the other one asked to start based on risk stratification. There is no clear direct way as in point, arrow to say where we should be going. Every time the guidelines update, then the compass change its spot […] it's very hard to aim for a moving target like for cardiovascular disease, right. We are saying we want to lower the LDL by 50% first, then aim to be as low as and as close to 1 as possible. So what 1.1, 1.8, 1.9? They're all close to 1. Which one do you want, right, and whether less than one is it OK? There is also like the fact that when the patient is frail, then they have to withdraw statins, right? At what point do we do this? So there is always [ambiguity] - because it's a spectrum, it's very hard to kind of tell which ones … how far you want to intensify it." "[…] if you don't have a clear demarcation of, you know, … if we don't meet this target by when, then we should intensify the therapy then that will be easier for intervention."
			Inconsistency in Lp(a) practices across individual clinicians	Different clinicians may place different level of emphasis on Lp(a) testing and management, leading to inconsistent practices	C023: Due to the lack of guidelines, within the department itself there is no consensus, some consultants will screen everyone for Lp(a), follow up and send to the endocrine genetic clinic, others will not do anything
			Prescribers' comfort level with lipid-lowering therapy affect decision on combination therapy	There might be reluctance among doctors (especially non-cardiologists) to prescribe combination therapy	C007: Participant suggested that some doctors may not be comfortable with prescribing lipid-lowering therapy like PCSK9-targeted agents, or even ezetimibe.
			Institutional policies might be slow to adopt changes to Lp(a) management	Organisations might take a long time or be reluctant to adopt new guidelines	C031: Despite ongoing research and recent development like AHA publishing data on lowering Lp(a), it takes time to establish clear management guidelines and determine side effects of treatment
		Limited manpower and resources	High attrition rate among clinical staff	Participant describes the challenge of routine screening due to high staff turnover	C003: Junior doctors are rotating a lot and there are new doctors coming in every 3 months, making it difficult to educate all to consistently order Lp(a) screening
			Concerns about manpower and workload to support follow-up	There may not be sufficient manpower for follow up with those found to have	C018: Participant was concerned with additional workload and limited manpower resources to follow

			after increased testing	elevated Lp(a) levels after testing	up on patients after testing
			Patients with elevated Lp(a) requires counseling	Patients' lack of awareness of Lp(a) takes effort to address and might hinder care	C009: Patients might be unfamiliar with the test/Lp(a) and it takes time from clinic schedule to educate patients
			Potential issue managing patients in non-cardiology setting	There are possible issue managing patients in certain departments	C027: Participant did not foresee any challenge managing patients, save for possibly in non-cardiology posting (possibly due to policies)
			Difficulty testing patients in outpatient setting	There are challenges to testing Lp(a) in outpatient setting	C020: Some difficulty in testing patients for high Lp(a) in the outpatient setting: "when we do lp(a) in the ward we are able to get the results almost immediately within the next 1 to 2 hours […] then he probably needs the medications and needs to have follow up with the cardio clinic ya, which is still okay but if lets say it is in outpatient environment then that will be a bit hard to screen lp(a)"
	Challenges in practice	On-the-ground hindrances	Difficulties communicating implication of Lp(a) testing and management to patient	Explaining Lp(a) testing and follow-up management to patients might be challenging	C032: Participant felt held back from ordering more tests, having to explain and justify to patients why Lp(a) is tested despite lack of acceptance and out-of-pocket cost.
			Fast-paced working environment	Clinicians are busy working in a high-stress environment and may struggle to attend to Lp(a) screening given limited contact time	C003: Fast paced, stressful working environment at clinics makes it difficult to consistently check for Lp(a) for every patient. Clinicians may overlook ordering test or checking if test has been ordered after index hospitalization event, as they are under pressure to rush through clinics and patients during ward rounds
			Other works compete with Lp(a) screening	Clinicians may not pay attention to the little specifics e.g., checking/ordering additional Lp(a) screenings when faced with multiple concerns to attend to	C003: Clinicians are often faced with competing concerns "have problems remembering some of the other more common things already how do you remember Lp(a)?"
			Fatigue and stress	Stressed out clinicians are unable to cope with workload on Lp(a) testing	C003: It is not realistic to rely on the often busy and tired clinicians/MOs to consistently remember to check on Lp(a)
		Perceived clinical	Workload and awareness of role are potential barriers for	Workload and awareness of role are potential barriers for nurses to be	AH005: "it's the workload that is number one. When the workload is high, it may not have time to

		hierarchy influenced roles in clinical management	nurses to be involved in lipid management	involved in lipid management	really look into the blood ordering for the patient unless it's specifically written in the clinical clin doc note. I think that is the major barrier that I would foresee. Second thing is knowledge […] if they're not aware that they're supposed to do, of course, then they won't do. So I think this will be the two potential barriers,
			Doctors decide nurses' involvement in educating patient about Lp(a) testing	Nurses' involvement in educating patients about Lp(a) testing is contingent on delegation by doctors	AH001: "[…] if let's say the doctors feel that there's a need for nurses to be involved, you know, in education on that, you know, […]. If not, I don't think they will talk to patient in specifically testing for Lp(a)."
			Nurse reticence in reminding doctors to order Lp(a) test	Nurses do not see it as their role to remind doctors to order Lp(a) test	AH001: [interviewer] So those that should be checked [Lp(a) testing] but didn't check, so you would think that nurses play a role in like reminding or? Participant: "I would think that they [nurses] probably would not because nurses may not be well versed with this Lp(a), you know
			Contentious whether to initiate PCSK9-targeted agents in outpatient or inpatient settings	Contentious discussion on where best to initiate PCSK9-targeted agent treatment, outpatients or inpatients. Implications are that someone will have to counsel the patient about the medication, show them how to use, etc.	AH005: Participant responded cautiously - with an eye to minimise their own (inpatient) workload. Hence, 'pushing it to outpatients' but qualifying it by saying 'we should flag it/start the conversation at inpatients setting'. [The Interviewer proceeded to mention one advantage of starting it in inpatients, to which the participant reluctantly agreed was an advantage (almost a mono-syllabic response).]
			Pharmacists may not be able to convince patients to agree to their doctor's suggestion to start PCSK9-targeted agent treatment	Pharmacists may not be able to convince patients to agree to their doctor's suggestion to start PCSK9-targeted agent treatment. The scenario is that the treating team flags up the patient for PCSK9-targeted agent initiation, and then ask the pharmacist to counsel the patient to negotiate, convince the patient to take up the medication.	AH009: "the initial part I still feel the consultant or what should have more say, it is more of trying to sell the thing [to the patient]. Like I say, it's more of how our society is wired. The way that people think the doctor is always the expert already regardless; if its coming from them [doctors] first, then subsequently when we [pharmacists] go in, anyone else [e.g nurse] go in, then it adds value. We are basically reiterating the point." AND doctors need to introduce the pharmacist to the patient as part of the team, "They need to introduce us as part of the whole programme, we are just a value added

					partner.”
			Prescribing etiquette for junior clinicians regarding combination therapy	Medical officers generally have no reservation highlighting to registrar or consultant in the care team to consider combination therapy	C021: Based on guidelines, patients’ readings and current suboptimal management, participant will prescribe on their own for simpler cases and obtain clearance afterwards, or highlight it to seniors for prescription
	Patients’ resistance to testing and treatment	Financial concerns	Concern on cost of testing	Participants are unsure about the cost of the test but think that it might be a possible barrier	C031: Some patients might not be able to afford testing if there is resource constraint – consultant emphasizes that “it’s not $7.00 it’s $50 per test” as suggested elsewhere
			Concern on cost of treatment	Clinicians note that elevated Lp(a) management might be costly for patients and providers	C019: Elevated Lp(a) intervention would be PCSK-9 or medication which is pricey currently, though there might be incoming subsidies. Lower price might make it easier to convince patients
			Implications on insurance coverage	Lp(a) testing result might affect patients’ insurance eligibility/premium amount and hinder their access to care	C010: Participant suggested that patients might be concerned about the insurance coverage for Lp(a) and its treatment
			“You need to meet your basic needs first, health usually takes a back seat”	PCSK9-targeted agents are expensive and unaffordable for some patients. Financial assistance will be needed.	AH004: “[…] but for patients who are needy and […] who cannot afford the therapy, their concerns is not only on the side effects and the benefits of the medicine […] will be whether I can afford the medicine at all. So if you're telling me that, oh, this medicine is gonna save my life, but I have to empty out my bank to use it and have no more money to eat afterwards, who will do that? Right? Because you need to meet your basic needs first you need to meet food, a place to stay, you know, and some money. So if you don't have all this, then health usually take a back seat.”
		Testing deemed undesirable	Testing might cause undue stress to patients	Routine testing might result in unwarranted stress and anxiety in patients	C031: Testing might cause emotional duress as young patient might grow up thinking there will be a heart attack due all the time, and after long term lifelong follow-up, treatment may not be available
			Lack of motivation to test for a genetic condition with no treatment	Patients might not see any point testing for elevated Lp(a) given it is genetically determined and currently no treatment is available	C030: Unlike ordering test for other indicator e.g., cholesterol, in the case of Lp(a), if informed that the condition is genetically determined with no therapy in market currently, patients might be resistant to

					the test as they do not see any purpose in doing so
			Patients' poor reception towards Lp(a) testing	Patients might be reluctant to test for Lp(a), thus discouraging clinicians	C032: Patients' "general acceptance of [Lp(a)] testing" holds clinician back from screening
		Treatment deemed too demanding	Resistance to long-term treatment	Patients might not be keen on long-term treatment following elevated results	C012: Participant believed that younger patients with no atherosclerotic cardiovascular disease may be reluctant to screen for Lp(a) as they may not be keen to take long-term medication
			Reservation towards relatively unfamiliar treatment	Patients might be less willing to take up uncommon therapeutic options such as PCSK9-targeted agents compared to other oral medication or lifestyle modification strategies, which are less effective at managing Lp(a)	C019: Half or more than half of patients are apprehensive of PCSK-9 and turn it down as treatment option due to its relative unfamiliarity compared to statins
			Injectable formulation is unpleasant	In the case of PCSK9-targeted agents, the treatment being an injectable is less palatable to patients	C027: All patients offered PCSK-9 decline treatment due to it being in injection format, oral medication is preferred
			Additional visits needed (if separate department is involved for follow-up) may discourage patients with time constraint from managing Lp(a)	Patients and their family members may struggle to attend additional clinic appointments due to time constraint and skip Lp(a) follow-up to minimize hospital visits	C027: Most patients and/or their family are busy working. It might be difficult to find time for additional hospital visits, especially if there are already existing general follow-ups to attend and Lp(a) follow-up is scheduled under a separate department like endocrine
		Poor compliance	Dosing schedule of PCSK9-targeted agents affects patient compliance	Dosing schedule of PCSK9-targeted agents e.g. inclisiran, affects patient compliance	AH009: "if you take something everyday, it becomes easier, it becomes part of your routine. If you take something like once a week, twice a week, once a month, gets a bit odd la. Maybe the 6 months it's not too bad because you come to clinic, we jab you. It is not like you take at home and then you [have to] remember when is the date you jab. […] you know your appointment date, we jab you today. That at least we got some control. If it's something like every – every 2 weeks you jab one time, every month you […] jab two injections. The other day I had one patient who actually he can't remember, then he omit. Then after that he confused himself. And then he create a diary but then he forget to chart. […] in the long run right, if one guy has the problem, out in the community, say in a thousand

					people, how many will have the same problem as well?
			Conflicting advice from doctors regarding target lipid levels affects patient compliance	Patient's compliance with lipid-lowering medications affected by conflicting advice from doctors regarding target lipid levels	AH004: "GPs are telling them, you know, lipid of this I think is it 200 or 100 LDL, it's OK you know you have met the target, but the target is for a normal person, it's not for a patient who has a high ASCVD risk score. […] patient also don't know what their own target is. If they don't know their target, they go to a different doctor, some doctor tell them this is good, some doctor tell them it's bad and they have a - they don't trust the healthcare system anymore, right?"
			Poor medication compliance precludes therapy intensification	Clinicians believe that poor compliance to existing medication should be addressed before intensifying treatment or declaring treatment failure	C027: Most patients prefer to reduce number of medications taken as it improves compliance, rather than adding on more for combination therapy
Enablers of Testing and Follow-up	Stronger clinical evidence base	Linkage to CVD	Strong evidence on the association of elevated Lp(a) with CVD risk can encourage testing	Clinicians may be convinced to test for Lp(a) on a wider scale given evidence that Lp(a) is associated with increased CVD risk	C004: Frequency of Lp(a) testing has increased in the past 1-2 years partly due to evidence showing Lp(a) as one of the risk factors for atherosclerotic diseases
		Benefits of testing	Evidence showing Lp(a) testing will lead to better patient outcome can facilitate wider adoption of Lp(a) testing	More evidence to prove that Lp(a) testing improves patient's outcome will improve awareness among physicians and increase Lp(a) testing.	C017: Clear and proven evidence that Lp(a) testing can lead to better outcomes will lead to more widespread testing of Lp(a)
			Local data demonstrating benefits of Lp(a) measurement may lead to better uptake	Local data on outcome of Lp(a) screening may help emphasise the importance of Lp(a) measurement.	C004: CGH-based database can be built to convince clinicians and patients of Lp(a) screening importance using local data on outcomes
	Institution-level efforts to support Lp(a) testing & management	Educational opportunities	Educational initiatives for HCPs	Participant highlight the importance of education such as presentation, workshop, case-based discussion, to increase awareness of Lp(a) among healthcare professionals	C016: as Lp(a) is getting more prevalent, educational seminars would be helpful to help doctors understand what to do in cases of high Lp(a) and what are the inclusion/exclusion criteria for clinic referral
			Education for clinicians on how to interpret Lp(a) results and what to do next	Guidance/education needed for clinicians on how to interpret Lp(a) results and the subsequent clinical management of the patient.	AH002: "[…]even if it's there, the results, […] some education needs to be done for them [clinicians] to know how to interpret it […] because [for example] nowadays the doctors just order, order, order renal

					panel but then potassium is low, nothing gets done about it.”
			Checklist of points for patient counselling on PCSK9-targeted agents	A checklist to aid pharmacists with patient counselling on treatment with PCSK9-targeted agent	AH003: “Then we can have on the pharmacist counselling point side, we can have our own checklist so whether patient is agreeable, whether patient feels confident to be able to inject the medication and then whether the patient can be compliant to it”
			Preparedness of nurses to field questions from patients about Lp(a) testing	If nurses were to be involved in educating patients about Lp(a) testing, nurses need knowledge to be able to answer frequently asked questions e.g. the necessity of the test, the cost, etc.	AH001: “I think they [nurses] will probably be asked by the patients, you know, why we need, alright, and how much it costs, you know, is it necessary? Yeah. So I think these are the potential questions that probably patient will ask and nurses […] should be told how do they answer for that questions that the patient asked lah. That's important. And then how often do you test my Lp(a), is it just one off or do you think I have to do it every few months or yearly […] nurses may be put in a fix, you know, if they do not know how to answer the question.”
		Standardised workflow	Standardised pathway for testing may encourage testing in practice	Lack of standardisation among physicians about Lp(a) testing presents a barrier, which can be countered by incorporating screening into care pathway, MI guideline for inpatient	C030: Lp(a) needs to be incorporated into pathways to increase awareness and build a culture even among junior staff, so the procedure would be second nature and automatically followed eventually
			Well established guideline can improve management of elevated Lp(a)	A clear guideline may allow clinicians to easily follow through for better management	C029: A guideline or a set of protocol is needed for a clear yes/no answer on inclusion-exclusion criteria when it comes to cardio patients with ACS issues. Lp(a) should be integrated into part of a workflow, coupled with clearer understanding it will promote people to actively think of Lp(a) and look out for patients who fit inclusion criteria to test
			Case managers are natural partners for patient education of Lp(a) testing	Involve case managers in implementing patient education of Lp(a) testing instead of all nurses as they already talk to patients about lipid-lowering medications and lifestyle modifications. Case managers also serve as point of contact for	AH001: “So the thing is that whether it will be good to involve all nurses, you know, or just case manager in particular, I would think that those that is required to talk to patients regarding lipid lowering drugs, you know or ways of [lifestyle] modifications, you know, then I think the case [manager] will be good.

				patients post-discharge.	
			Nurses can remind doctors to order Lp(a) testing if prompted by clinical pathway	If Lp(a) testing is included in a clinical pathway, and nurses notice that it has been missed by doctors, then nurses can remind doctors to order the test.	AH005: the inclusion of Lp(a) testing in a clinical pathway serves as a prompt to remind nurses to flag doctors to order it (if missed) and/or the doctor will see it on the clinical pathway and order it themselves.
		Accessible testing and treatment options	Subsidies for Lp(a) tests and treatment may improve detection rate	Subsidies are needed to alleviate financial burden and better convince patients to test for Lp(a)	C019: Elevated Lp(a) intervention would be PCSK-9 or medication which is pricey currently, though there might be incoming subsidies. Lower price might make it easier to convince patients
			Improved access to testing and follow-up facilities may increase the use of Lp(a) test	Testing will become more prevalent as its availability improves	C032: Availability and affordability of testing can be improved.
			Availability of treatment for high Lp(a) will increase the use of Lp(a) test	HCPs may be convinced to test for Lp(a) in a wider scale given a treatment targeting elevated Lp(a)	C026: If strong treatment to lower Lp(a) or preventive measures are available it will not be difficult to convince patients
			Partnership with healthcare providers in the community for long-term care of patients post-AMI	Description of how the nurse's acute hospital can work with/ weave together a network of community healthcare partners, including telehealth modalities of care for long-term care, monitoring, and follow-up of patients post-AMI.	AH006: "But we do have our colleagues right, who work very closely with […] our telecarer, who calls the patient to reinforce and also our community nurse, our primary care partners; probably that will be our- […] what I can foresee is we can build that components in our partners, right. Probably we can't see everybody in the whole community, but there's the whole HealthySG, right, the initiatives and the intent. So we can probably build that components in our collaboration when we work with our primary care partners. So then they will help us to monitor them."
			Increasing Lp(a) testing and management needs a ministry-level top-down approach		AH009: "I do not know what is MOH stand, I always feel that things like this right, you probably need to go up to that level for it to trickle down into something. Just like in the UK, insicliran is part – PCSK9, insicliran is also part of their formulary treatment, they are also pushing for it. How it got started is because somebody did a cost-analysis study, they are all big on these studies, they presented it at their parliament and so on and then it got things moving, start from there then it led

					down.”
		Technological innovations	Improvement to current system interface may facilitate Lp(a) testing	Improvements to the existing medical record system can ensure consistency in deploying screening protocol in practice e.g., reminder pop-up screen can be programmed in response to pre-set criteria for Lp(a) testing	C016: A visual difference to highlight elevated Lp(a) result will be helpful. Currently display of Lp(a) score in CGH SCM is always in black by default and there is no difference for high versus normal level, some may not remember that the cutoff is 50 especially if they are not in cardiology. "if it's more than 50, then it should be red wah. So people will know that it is a positive risk enhancer”
			Technology enhancement for patient care	Leveraging technology such as health apps may improve patient care	C008: Using apps/video to teach patients about Lp(a) and its management
			Usage of certain Lp(a) assays	Certain Lp(a) assays may offer more accurate results and should be offered for clinical use; HCPs may be less inclined to order test if given the choice of other assays	C030: Lp(a) assays used in private sector often has an upper threshold, beyond which consultant cannot tell exactly how high the measurement is, as such it is not ideal for clinical use. Lp(a) is directly measured in nmol/L in CGH and thus does not face this issue
			Inclusion of Lp(a) test into existing panel screening	Ordering Lp(a) test can be made more convenient to clinicians by coupling with existing tests	C023: currently only HbA1c and lipid panel test are ordered, Lp(a) can be 3rd marker
	Efforts to enhance literacy among non-HCPs	Among patients	Patient education efforts	There is a need to raise awareness on Lp(a) testing and management among patients and family members	AH007: suggested modalities for creating publicity and awareness e.g. social media, facebook posts, involve HPB, even simple messages about cholesterol, LDL, HDL, and include Lp(a) too.
			Explanation about Lp(a) level on health report can help with patient understanding	Additional information on Lp(a) can be provided to patients on health report to improve understanding	C008: Explanation about Lp(a) level on health report can help patients gain understanding about this blood test and its significance
		Among general population	General public awareness of the importance of Lp(a) testing	Advocating for routine screening among cardiovascular population is a possible solution to improve high Lp(a) detection rate	C009: It is important to raise awareness among general public, lipid clinic can help educate patients on the significance of Lp(a) testing
			Health literacy can be improved among general population to raise awareness of Lp(a)	Medical literacy can be improved to raise awareness of Lp(a) among general population	C021: In alignment with current HealthierSG policy, medical literacy can be improved to increase understanding on Lp(a) among general population as the subsequent stage after increasing awareness among HCP

Ideal system to support patient management	Context and setting of Lp(a) testing	Index patient testing	Appropriate setting to test for Lp(a)	Where the test is ordered (inpatient or outpatient) depends on when the participants encounter the patient	C003: Lp(a) testing should be done during inpatient stay prior to discharge for those hospitalized for ACS. Outpatient clinic testing is only for cases of high LDL in young patients or at post-AMI clinics 2 weeks after discharge. Ideally inpatient should be the main focus, primary care should be prioritized to prevent first event as secondary prevention is often too late.
			Timing of Lp(a) testing is not critical	Participants do not believe there is a critical timeframe for conducting Lp(a) tests in specific clinical situations or index events such as AMI	C030: Participant will test for Lp(a) at point of admission together with other blood tests and does not wait for any amount of time or have cutoff e.g., 3 months post-event. Though MI is potentially inflammatory condition and may affect readings, from participant's experience if Lp(a) is high then it will still be high at point of index event
			Repeat Lp(a) testing is not necessary	Participant believes that patients with high cardiovascular risk or previous MI should have Lp(a) measured "at least once in their lifetime" and for now, repeat testing is unnecessary	C016: Participant identified no evidence that trending Lp(a) makes a difference in overall outcomes, one-time measure is sufficient to counsel on accelerated atherosclerosis risk, diet and LDL control
			Possible use case for repeated Lp(a) testing	Participants describe different scenarios for repeated Lp(a) testing	C030: A second test is needed if Lp(a) is in the grey zone or there are factors contributing to elevated Lp(a) e.g., concurrent infection – to repeat once infection is cleared
		Cascade testing	Uncertainty about cascade testing for high Lp(a) levels	Participants are unaware or uncertain of cascade testing as a follow-up to elevated Lp(a) level in patients	C016: Participant is not familiar with cascade testing, other than that Lp(a) is a risk enhancer and cascade testing would be recommended if familial hyperlipidaemia is concerned, but high Lp(a) result on its own would not be enough for cascade testing recommendation
			Lack of recommendations and guidelines on cascade testing	Participants are aware of cascade testing, but are uncertain of management and follow-up due to lack of guidelines on cascade testing	C031: There is currently no guideline on referral in cases of borderline elevated Lp(a) (80-90), even though at that level patients would have increased cardiac risk, MACE events and AMI/IHD may be clearly present "right now in cardiology we don't have a cascade screening programme. We don't have research, you know, enablers" (unspecified)
			Possible use case for cascade	First degree relatives of patients with	C030: patients with elevated Lp(a) of above

			Lp(a) testing	familial hypercholesterolemia; history of premature CV events; high Lp(a) should be referred for cascade testing	50mg/dL or about 125nmol/L, especially if index case already has atherosclerotic, cardiovascular disease or stroke, should be tested
	Selection of patients for Lp(a) testing	Inclusion/exclusion criteria	Appropriate patients for Lp(a) testing	Participants describe scenario or rationale to justify Lp(a) testing. Some believes all CV patients (MI or recurrent CV events) or even all cardio patients including non-ACS conditions like AF should be tested as precaution, others believe inclusion criteria should prioritise only a sub-population of ASCVD patients with no apparent risk factors/well controlled risk factors, younger patients (40-50), patients with FH history (also applicable for cascade testing)	C019: all incoming ACS patients typically have fasting lipid panel done as default, those found with high LDL or hypercholesterolemia should then be tested for Lp(a). Alternatively, those with physical symptoms of hypercholesterolemia such as xanthelasma or atheroma, patients with strong family history of IHD or CVD risk, young patients with ACS like STEMI, NSTEMI, UAP or even ECG changes indicative of early IHD but without significant risk factors (e.g., diabetes, high blood pressure, elevated LDL), or recurrent ACS/stent thrombosis/stent stenosis despite well controlled risk factor (compliant to medication or lifestyle modification) would raise suspicion of possible genetic underpinning. Patients with high ASCVD risk, premature CAD family history or admitted for MI should be tested
			Patients that do not need Lp(a) testing	Participants describe the reasons or scenarios where Lp(a) testing is not required.<br><br>Most identify old age (>80) to be exclusion criteria, some identified low risk profile patients (non-cardiac related symptoms, low LDL level, stable condition) or ACS patients with other apparent risk factors	C031: patients with chest pain without confirmed IHD e.g., heart failure, non-ischemic myocardiopathy, myocarditis; patients with established stable condition e.g., CABG 20 years ago without subsequent events should not be tested despite the push for screening
		Practical considerations on Lp(a) testing	Patients eligible for Lp(a) testing, but at risk of not getting captured by current practice	Participants describe why current Lp(a) testing procedure might not reach certain groups of eligible patients	C003: Ideally all patients with CAD should be tested, though currently only the AMI programme is being leveraged to capture patients and hence currently eligible patient pool is missing out on those not on any pathway: patients with only angina pectoris, those going for outpatient test, picked up abnormal stress test and came for a coronary angiogram.
			Pragmatic selection of	There might be potential issues	C014: Clear inclusion-exclusion criteria for testing

			appropriate patients for Lp(a) testing	identifying the correct demographic of patients for Lp(a) testing	are needed, there is a risk of false negative if everyone is screened. Consultant is unclear whether there's any benefit screening old patients with poorly controlled LDL or focus on young MI/ACS/high lipids patients
			Testing Lp(a) in patients with high LDL may improve high Lp(a) detection rate	Checking Lp(a) level for patients with high LDL is suggested as one way to improve high Lp(a) detection rate	C024: Screening patients with elevated cholesterol and family history of high cholesterol/familial hypercholesterolemia and routine screening of patients with CVD can improve detection rate for high Lp(a) and hypercholesterolemia
			Testing Lp(a) among patients in vascular department may improve high Lp(a) detection rate	Checking Lp(a) level for patients with vascular disorders may improve detection rate	C031: very large pool of patients in vascular department can be leveraged for select population screening, including any patient with systemic atherosclerosis e.g., stroke and PVD
	Post-Lp(a) testing follow-up	Clinic referral	Management strategies/clinical actions following Lp(a) testing	Participant discusses the different management strategies for Lp(a) results (excluding LDL goal which is covered under code 29)	C028: Participant refers elevated Lp(a) (>120) patients to lipid clinic and is hence unsure of any changes to management practices. For <120 cases, there is no change "just continue the usual aggressive statins and lifestyle modification". Borderline cases may receive counselling and be considered for lipid clinic referral and aggressive statins, though usually patients with atherosclerotic events should already be placed on such therapy
			Criteria for referral to lipid clinic	Participants describe the various thresholds to refer difficult cases to CGH lipid clinic e.g., patients with possible familial hyperlipidaemia based on Dutch lipid criteria; high risk patients with LDL >1.4mm/L, especially for compliant patients after medication (statin therapy, PCSK9-inhibitors, or inclisiran) does not work well, patient with complications associated with dyslipidaemia (e.g. pancreatitis associated with hypertriglyceridemia), statin intolerant, patient reluctance to starting PCSK9-targeted agent	C009: Patients whose lipid levels remain high despite optimal multiple treatments, patients unable to tolerate statins, ambivalent about PCSK-9 inhibitor or inclisiran injection, can be referred to endocrine for counsel

			Elevated Lp(a) threshold for referral to lipid clinic	Participants discuss the different thresholds considered to be elevated Lp(a) for referral	C032: Referral is mainly for the ones with either very high Lp(a) (>125) It depends on which threshold cutoff is used – original ESC guideline uses 50 and 125 though latest guideline uses a higher cutoff for greater predictive value
		Combination therapy	Scenarios to start combination lipid lowering therapy	Participants described the scenario when combination therapy is necessary and the step-wise approached of prescribing lipid medications	C014: combination therapy is standard of care especially for IHD patients with hyperlipidemia and proven arteriosclerosis, as statins together with ezetimibe and PCSK-9 are needed together to achieve the lower than typical LD target
			Opinion on LDL targets in relation to Lp(a) level	Participants generally believe that LDL target following therapy is not influenced by Lp(a) level	C010: LDL target will not change as the patients whom the participant will test Lp(a) for are already at high risk and therefore should already be aggressively treated
			Lower LDL targets for patients with high Lp(a)	Participants believe that LDL target should be lower for patients with high Lp(a)	C002: Participant was unsure and there nothing “cast in stone”, since statin therapies technically don't lower Lp(a) significantly and there’s no proven treatment targeting Lp(a), but in cases where patients’ LDL is well controlled, yet still have active ASCVD, best option would still be using LDL-lowering therapy to bring LDL down further by 30-50%
			Specific LDL targets for patients	Participants specify targets in different groups of patients	C014: guideline directed therapy usually aimed for <1.4, which is difficult to achieve in practice and so those without very extensive heart disease may aim for 1.8. LDL goal is set to be as low as possible for higher risk patients with multiple IHD episodes or very challenging IHD cases
			Understanding the barriers from patient’s perspective about PCSK9-targeted agent treatment	Before initiating PCSK9 treatment, the prescriber and/or pharmacist needs to elicit and understand patient’s barriers for agreeing/accepting this treatment	AH004: “[…] it is about building rapport with the patient to understand what their concerns are about […] Of course, we do have to go through all the side effects and background of the medicine. But that's not what the patient needs. Actually they can have all the information, but most of the time they want to want someone to tell them whether this is good for them and whether you know, their pocket will get a hole after using it or not. So really, you also have to understand the patient to know

					whether they have any financial issues, what's their barriers from starting the medicine and also to break down how we can help them get this medicine if they are willing to do it? Yeah. So for prescribing, it's not just about counselling or the side effects and the pros, it's really also to find out where the barrier - what kind of barriers the patient have to starting this medicine and of course you can say that a lot of patients just say I don't want new medicines but sometimes some patients really want the therapies, but they just don't have access to it and this one will probably have to clear up as a prescriber or the pharmacist. In fact, if required, the MSW have to step in."
	Manpower/ Services to support Lp(a) management	Role of clinicians at different levels	Seniority level of clinicians required to follow up and manage high Lp(a)	Clinicians from multiple specialties regardless of seniority are suggested as suitable to adequately manage Lp(a). MO and registrar or senior residents may run clinic and attend to patients if sufficient training is provided	C013: anyone regardless of seniority as more people know how to manage this condition
			Specialty to follow-up and manage high Lp(a)	Cardiologists and endocrinologists are suggested as suitable disciplines to manage patients with high Lp(a)	C025: MO expressed that the preferred specialty depends on whether management labels hyperlipidaemia as a genetic, endocrine, or generic issue (since "everyone can have high cholesterol"). If former - geneticists should be involved and trained in counselling families; if latter – endocrine is already involved in following up through the lipid clinic.
			Healthcare services to follow up and manage Lp(a)	Participants discuss which level of healthcare services are suitable to follow up on high Lp(a) patients	C014: given the lack of Lp(a) treatment options, only follow-up strategy is atherosclerosis treatment and early surveillance, which can be provided at polyclinics if clinicians know what to look out for
		Role of pharmacists and allied health professionals	Role of pharmacists	Pharmacists can play a role in educating patients about medications for lipid management, medication dosage titration and answer patients enquiries about Lp(a). Clinicians	AH004: [to convince patient to consider PCSK9-targeted agent treatment] "I guess if the doctors are really keen for the patients to start, they can actually probably kind of prime them and tell them that, hey, I think you're suitable for this medicine,

				believe pharmacists are best suited for explaining medication to patients, specifically regarding administration of PCSK9 inhibitor injection and statins side effects; and also may help in limited capacity to run clinics and protocolise management	why don't I refer you to my lipid pharmacist? Then we can go through the medicines from there and then the pharmacist who runs the lipid clinic can have more time to do the counselling and negotiation with the patient."
			Role of allied healthcare professionals in general	Allied healthcare professionals can be involved in ward rounds and clinic operations and are an important part of the team to deliver holistic care to patients and reinforce the instructions and advice for better compliance.	C032: Everyone has a role to repeat the same instructions to patient to ensure compliance "to be honest, a lot of it is repetition. I think if everyone sing the same song and on the same page, then patients are more likely to take it seriously"
			Role of dietitians	Dietitians primarily focus on dietary education to help patients better control their eating habits as part of lifestyle management strategy to lower lipid levels	C031: Dietitians should be involved, as diet management is part of lifestyle modification although consultant doubts dieting can lower LDL and is unsure if it might lower Lp(a) level
